# New Gluten-Free Extruded Snack-Type Products Based on Rice and Chickpea and Fortified with Passion Fruit Skin: Extrusion Cooking Effect on Phenolic Composition, Non-Nutritional Factors, and Antioxidant Properties

**DOI:** 10.3390/molecules30061225

**Published:** 2025-03-09

**Authors:** María Ciudad-Mulero, Erika N. Vega, Patricia García-Herrera, Samuel Fernández-Tomé, Mercedes M. Pedrosa, Claudia Arribas, José De J. Berrios, James Pan, Priscila Leal, Montaña Cámara, Virginia Fernández-Ruiz, Patricia Morales

**Affiliations:** 1 Departamento de Nutrición y Ciencia de los Alimentos, Facultad de Farmacia, Universidad Complutense de Madrid (UCM), 28040 Madrid, Spain; erinino@ucm.es (E.N.V.); patrigar@ucm.es (P.G.-H.); sfernandeztome@ucm.es (S.F.-T.); mcamara@ucm.es (M.C.); vfernand@ucm.es (V.F.-R.); patricia.morales@farm.ucm.es (P.M.); 2 Departamento de Tecnología de Alimentos, Instituto Nacional de Investigación y Tecnología Agraria y Alimentaria (INIA-CSIC), 28040 Madrid, Spain; mmartin@inia.csic.es (M.M.P.); arribas.claudia@inia.csic.es (C.A.); 3 United States Department of Agriculture-The Agricultural Research Service-Western Regional Research Center (USDA-ARS-WRRC), Albany, CA 94710-1105, USA; jose.berrios@usda.gov (J.D.J.B.); james.pan@ars.usda.gov (J.P.); priscila.alves@usda.gov (P.L.)

**Keywords:** pulses, gluten-free, extrusion, phenolic compounds, non-nutritional factors, antioxidant activity

## Abstract

The incorporation of pulse flour into gluten-free extruded snacks based on cereals improves the functional properties as well as the nutritional value of these types of products. The aim of this study was to investigate the changes induced by the extrusion process on the functional properties in terms of the concentration of total phenolic compounds (TPC), phenolic families (hydroxybenzoic acids, hydroxycinnamic acids, and flavonols), and non-nutritional factors (inositol phosphates and trypsin inhibitors) of extruded snack-type products developed from novel formulations based on rice-chickpea flours and fortified with different percentages of Fibersol^®^ and passion-fruit-skin flour. The in vitro antioxidant activity of the studied formulations was evaluated to explore their potential for developing sustainable snack-type products with added functional value. The results demonstrated that extrusion treatment caused a statistically significant (*p* < 0.05) decrease (12–30%) in TPC. Despite this reduction, the extruded formulations preserve an interesting content of these compounds, with hydroxybenzoic acids being the majority in the analyzed formulations. The extrusion process maintained or decreased the content of phytate and total inositol phosphates in samples fortified with passion fruit and Fibersol^®^. A significant reduction (*p* < 0.05) of trypsin inhibitor activity (between 86.7% and 95.8%) was observed when comparing extruded samples to their raw counterpart. The antioxidant activity in vitro of the formulations was assessed. The results obtained by the Folin–Ciocalteu method indicated that extrusion caused a decrease in the antioxidant activity of 50% of the analyzed samples, while in the others, no changes were observed. DPPH and FRAP assays tended to demonstrate an increase in antioxidant activity. In general, the highest values were obtained by applying the DPPH method. Additionally, the effects of the ingredients used for fortifying the formulations were investigated. The results highlighted the complexity of the analyzed formulations, revealing that their composition is influenced not only by the presence of Fibersol^®^ and passion fruit but also by the interaction between these two ingredients.

## 1. Introduction

Gluten-free products often have a higher glycemic index and lower nutritional value compared with gluten-containing products. To resolve this problem, the food industry considers the use of a mixture of several ingredients, looking for different types of starch or gluten replacements that offer not only better nutritional properties but also good taste, appearance, and shelf life [1]. The incorporation of pulse flour into extruded snacks based on cereals is an interesting strategy that significantly improves the functional properties as well as the nutritional value of snack-type products [2]. This fact is of great interest to the food industry, as this sector is continually innovating to satisfy the exigent demands of consumers, who search for novel food products with suitable organoleptic characteristics, proper nutritional profiles, and added functional properties. In addition, the commercialization of snack-type products in the market has increased over the years, and it is particularly noted in the gluten-free sector to satisfy the demands of the celiac population [3].

During the last few years, the use of extrusion for developing gluten-free products has been extended. Extrusion is a versatile technology commonly used in the food industry. The process involves feeding raw materials into the extruder barrel, where they are subjected to the rotational movement of the screw(s) along with increased pressure, high temperatures, and shear forces for a short duration. These conditions induce changes in the content of bioactive compounds and antioxidant properties, leading to the inactivation of non-nutritional factors [4,5,6].

Pulses are gluten-free foods with high nutritional value. They contain phenolic compounds, also known as polyphenols, which are bioactive secondary plant metabolites that contribute to the color and sensory characteristics of the seed. These compounds provide several biological properties, most of them related to their high antioxidant capacity. Phenolic compounds include a wide range of molecules classified into different groups according to their structure. In legumes, the main groups of polyphenols are phenolic acids, flavonoids, and condensed tannins [7,8].

Additionally, pulses contain minor constituents known as antinutrients or non-nutritional factors, which can have bioactive effects depending on their concentration and interaction with other dietary components [4,9]. This is the case of inositol phosphates and trypsin inhibitors. The first is responsible for a decrease in mineral bioavailability. However, inositol phosphates have a positive effect on human health, as they reduce the bioavailability and toxicity of heavy metals, reduce the risk of kidney stone development, and modulate hypercholesterolemia [10]. On the other hand, trypsin inhibitors reduce the biological activity of trypsin; however, they are suggested to have some health benefits, including the prevention of various types of cancer [11,12].

Pulses are notable not only for their nutritional value and functional properties but also for their potential to contribute to the development of sustainable food systems. Sustainability is an increasingly important concern for society. Pulses enhance soil fertility without the need for nitrogen fertilizers, and they require less water to grow compared to other crops. Furthermore, incorporating fruit by-products such as seeds, peels, leaves, and unusable pulp as functional ingredients in the production of extruded snacks made from pulses can improve their functional quality. This approach also helps minimize food waste and reintegrates agro-industrial by-products into the processing chain, supporting a circular bioeconomy [13,14]. One of the fruit by-products that has been used as a functional ingredient in the last few years is passion fruit skin. Passion fruit is widely consumed in Latin countries and has seen increasing global popularity in recent years. Most of the fruit is processed by the food industry for juice production, resulting in several thousand tons of seeds and peels (45–52% of the fruit) as byproducts. These passion fruit peel byproducts are rich in vitamins, minerals, polyphenols, and dietary fiber, making them highly suitable for use as ingredients in new product formulations, where they act as functional ingredients [15,16], as is the case of the present study.

Additionally, extruded snacks made from pulses can be enhanced by adding ingredients like Fibersol^®^, a highly digestion-resistant corn-maltodextrin that is non-viscous and water-soluble. This ingredient has interesting technological properties for developing gluten-free products. In addition, Fibersol^®^ stimulates the production of satiety hormones, thereby improving feelings of fullness/satiety in humans [17,18].

During the last years, the interest in developing gluten-free formulations based on mixtures from pulses and cereals and fortified with different sources of bioactive compounds (polyphenols, dietary fiber) has increased. For instance, there is scientific literature that deeply analyzes the biochemical composition of extruded rice/white bean gluten-free fettuccine fortified with whole carob fruit flour [19], gluten-free bread based on rice/field bean flour enriched with pomegranate seed powder [20], or gluten-free bread based on corn starch, rice flour, and lentil flour enriched with green coffee parchment [21].

Samples analyzed in the present work have been previously characterized from the point of view of their carbohydrate fraction [17]; thus, the aim of this study was to investigate the changes induced by the extrusion process on the functional properties of extruded snack-type products developed from novel gluten-free formulations made from rice and chickpea flours fortified with Fibersol^®^ and passion-fruit-skin flours. Specifically, we focused on the concentration of total phenolic compounds, various phenolic families (including hydroxybenzoic acids, hydroxycinnamic acids, and flavonols), and non-nutritional factors such as inositol phosphates (IPs) and trypsin inhibitors (TIs). Furthermore, we evaluated the in vitro antioxidant activity of the formulations to assess their potential for developing a sustainable snack product with enhanced functional value.

## 2. Results and Discussion

### 2.1. Total Phenolic Compounds and Phenolic Families

In the present study, total phenolic compounds, as well as the content of hydroxybenzoic acids, hydroxycinnamic acids, and flavonols, were analyzed by QUENCHER methodology, and the results are featured in Table 1.

The total phenolic content in raw analyzed formulations varied between 8.53 and 14.28 mg GAE/g (samples CF#3 and CF#2, respectively). Among raw samples, it was observed that the untreated sample, which exhibited a significantly lower (*p* < 0.05) concentration of total phenolic compounds (CF#3), was formulated with 83.78% of rice-chickpea blend, 5% of passion fruit, and 5% of Fibersol^®^. It was noticed that raw formulations that include a lower percentage of passion fruit (CF#3, CF#6, and CF#9) showed lower total phenolic content compared to the others. Moreover, a significantly higher (*p* < 0.05) level of total phenolic content was found in samples CF#2, CF#5, and CF#8, all of them characterized by containing 12.5% of passion fruit in their formulation. In general, the blends under study had a higher content of total phenolic compounds than those reported by other authors who have previously studied different flours based on pulses and/or cereals [22,23,24]. This fact suggests that the incorporation of passion fruit skin flour in this type of formulation is a good strategy to increase their content of phenolic compounds, allowing the obtention of food products with added functional properties. Moreover, in view of the obtained results, the addition of a percentage of passion fruit close to 12.5% could be the optimum for this purpose.

The extrusion treatment caused a statistically significant (*p* < 0.05) decrease in total phenolic content. The obtained values for extruded samples ranged from 6.32 to 11.30 mg GAE/g in samples EF#4 and EF#2, respectively. Despite this reduction, which was around 12–30%, the extruded formulations preserved an interesting content of total phenolic compounds, which was higher than that reported by Morales et al. 2015 for lentil flours fortified with different proportions of wheat bran, corn fiber, apple fiber, and Nutriose^®^ [22]. The scientific literature reported that extrusion treatment is usually associated with a reduction in phenolic content due to the thermal impairment of their structures when subjected to high temperatures and/or polymerization [25]. For instance, this effect has also been observed in cassava-soy composites enriched with grape pomace [26], in expanded snack products based on corn grits and fortified with brewer’s spent grain, sugar beet pulp, and apple pomace [27], or in maize extrudates [28].

Phenolic acids can be grouped into two categories, namely hydroxybenzoic and hydroxycinnamic acids. It has been reported that these compounds possess antioxidant and anti-inflammatory properties [29]. As shown in Table 1, hydroxybenzoic acids were the majority in the analyzed formulations based on rice and chickpea, and their content varied between 1.91 (CF#5) and 5.35 (CF#2) mg GAE/g and between 1.62 (CE) and 3.94 (EF#9) mg GAE/g in raw and extruded formulations, respectively. These values were similar to those found in lentil flours fortified with different sources of fiber, where raw and extruded samples showed a content of hydroxybenzoic acids of 1.88–2.99 mg GAE/g and 3.15–5.76 mg GAE/g, respectively [22]. After extrusion treatment, the main tendency consisted of a significant decrease (*p* < 0.05) of the hydroxybenzoic acid content, maybe due to the high temperature applied that caused their decomposition [30]. However, in the case of samples EF#5 and EF#9, a significant increase (*p* < 0.05) of the hydroxybenzoic acid content was observed because of extrusion. The content of hydroxybenzoic acid in EF# 5 and EF# 9 increased by 23.04% and 83.26%, respectively, compared to that of CF# 5 and CF# 9. Although it is known that extrusion treatment greatly affects the composition of phenolic acids in cereals, it has been observed that the effect of this technological process is significantly different depending on the food matrix and extrusion conditions [30].

Regarding the content of hydroxycinnamic acids, the concentration of these compounds in raw samples ranged from 0.28 to 0.59 mg FAE/g in formulations CF#6 and CR, respectively. Statistically significant differences (*p* < 0.05) were found among the studied raw formulations. It was noticed that the control formulation (CR) had the highest content of hydroxycinnamic acids, followed by samples CF#1 and CF#7 (both with the maximum percentage of passion fruit). The cited samples CF#1 and CF#7 were not statistically different compared to sample CF#4. By contrast, the lowest content was found in sample CF#6 (formulated with 81.25% of rice-chickpea blend, 5% of passion fruit, and 7.5% of Fibersol^®^). No significant differences (*p* > 0.05) were found between samples CF#6, CF#5, and CF#9, attending to their content of hydroxycinnamic acids. The extrusion process caused a significant (*p* < 0.05) decrease in hydroxycinnamic acid content. In this regard, it has been reported that the effect of the extrusion treatment on the phenolic compounds content depends on the processing conditions (temperature, moisture, screw speed) and the ingredients of the food matrix (pulses, cereals, pseudocereals, fruits, vegetables, seeds, herbs, or mixtures of them) [25]. This important fact could explain why other authors have previously found an increase in hydroxycinnamic acid content in different food products after extrusion cooking [22].

As previously indicated, in the present study, the phenolic family represented by flavonols was quantified (Table 1). The concentration of these compounds in raw samples varied between 0.067 and 0.140 mg QE/g (formulations CF#2 and CR, respectively), while in the case of extruded samples, they ranged between 0.084 and 0.138 mg QE/g (formulations CE and EF#8, respectively). It has been reported that the content of flavonol in chickpeas ranged from 0.008 mg/g to 0.242 mg/g [31], and our results are in accordance with these observations. Table 1 shows that as a consequence of extrusion treatment, no significant changes (*p* < 0.05) were observed in samples #4, #5, and #9. Nevertheless, a significant increase (*p* < 0.05) in flavonol content was appreciated in formulations codified as #1, #2, #3, #6, and #8, while it was significantly decreased in sample EF#7, compared with its corresponding raw counterpart. It is noted that flavonols in foods are usually found as O-glycosides, which improves their thermal stability during flour processing. Moreover, thermal processing, such as extrusion, also affects the release of phenolic compounds due to the disruption of the food matrix. This fact could result in flours with a higher content of flavonols [32]. However, the contrary effect of extrusion has also been reported; Morales et al. (2015) observed that flavonols suffered significant (*p* < 0.05) reduction after the extrusion treatment [22]. These findings suggest that the effect of thermal treatment on flavonols is highly dependent on the type of food matrix.

### 2.2. Inositol Phosphates and Trypsin Inhibitors Activity

Table 2 shows that the total inositol phosphate content of analyzed samples ranged from 2.03 (formulation CF#7) to 2.97 (formulation CF#1) and from 1.68 (formulation EF#7) to 3.52 mg/g (formulation CE) in raw and extruded samples, respectively. Regarding individual compounds, inositol hexaphosphate (IP6), also known as phytic acid or phytate, was the prevalent isoform in all analyzed formulations, followed by inositol pentaphosphate (IP5) and inositol tetraphosphate (IP4). This tendency was previously observed in several scientific research studies focused on analyzing the content of inositol phosphates in different pulse flours [4,23]. In the present study, significant differences (*p* < 0.05) were found concerning IP5, IP6, and total inositol phosphate content in raw analyzed samples. Moreover, the control raw formulation, as well as untreated formulations with the lowest percentage of Fibersol^®^ showed a significantly higher (*p* < 0.05) content of IP6 and total inositol phosphates. This fact could indicate that Fibersol^®^ interferes with the extraction of inositol phosphates, making it difficult. However, the inclusion of passion fruit in analyzed formulations also impacts the content of IP6 and total inositol phosphates, as discussed in Section 2.4. of the present manuscript, showing that the content of IP6 and total IP is influenced not only by the presence of Fibersol^®^ and passion fruit but also by the interaction of both ingredients.

Extrusion treatment did not result in statistically significant changes in IP4 or IP5 content (*p* > 0.05). Nevertheless, the content of IP6 and total inositol phosphates were significantly increased (*p* > 0.05) as a consequence of this technological process in the case of the control raw formulation based on chickpea–rice. In the rest of the samples, which were fortified with passion fruit and Fibersol^®^, the extrusion process maintained or decreased the content of IP6 and total inositol phosphates. These results suggested that the enrichment of chickpea–rice flours by incorporating passion fruit and Fibersol^®^ conducted a reduction of inositol phosphates, which are considered antinutrient compounds as they reduce the bioavailability of some minerals [23]. Other authors have evaluated the effect of the enrichment with fruit by-products of different food matrices based on grains on the inositol phosphates content. Specifically, de Toledo et al. (2019) developed cookies with partial substitution of wheat flour by fruit by-products (melon peel, apple endocarp, and pineapple central axis) and observed that these replacements were associated with lower levels of phytate compared with the control. Moreover, it was found that the incorporation of pineapple by-product and melon by-product in cookies improved zinc and iron absorption, respectively [33]. In this line, with the aim to promote the consumption of cereal-pulse flours, Udomkun et al. (2019) evaluated the composition of different formulations, which included maize, wheat, sorghum, sesame, and soybean flours, enriched with cassava flour, rice flour, or banana flour. These authors reported that the full replacement of wheat flour with banana flour caused a reduction in phytate content from 1.71 to 1.54 mg/g [34].

Trypsin inhibitors (TIs) are protease inhibitors found in raw pulse seeds. These compounds have been shown to hinder protein digestion and are considered non-nutritional factors since they can reduce the nutritional quality of pulse-based foods. However, pulses are not consumed raw. Instead, they are consumed after thermal treatment. In order to inactivate or reduce the TIs’ concentration of pulses below the threshold limits (1–1.5 TIU/mg) that allow them to be considered safe for human consumption, several thermal processes have been used [35]. The extrusion process has exhibited its effectiveness in achieving this purpose [4,23].

In the present study, the trypsin inhibitor activity (TIA) of analyzed formulations has been evaluated, and the results are shown in Table 2. There was a statistically significant difference (*p* < 0.05) between the majority of analyzed samples. Raw analyzed formulations contain between 2.47 (CF#5) and 6.21 (CF#2) TIU/mg. This range was similar to that previously found in lentil flour formulations enriched with nutritional yeast (3.50–4.32 TIU/mg) [4]. There were no significant differences (*p* > 0.05) between samples CF#5, CF#7, and CF#4, which showed values of TIA significantly lower (*p* < 0.05) compared to the rest of the raw formulations. Whether these results are related to the composition of the samples, it is observed that formulations CF#7 and CF#4 presented the lowest content of rice/chickpea flour (63.75% and 66.25%, respectively) and the highest proportion of passion fruit (20% in both cases). This fact suggests that the incorporation of passion fruit by-products could be effective in improving the nutritional quality of flours based on pulses. Other authors, who analyzed lentil flour formulations enriched with apple fiber, among other ingredients [9], have previously observed this positive effect attributed to the addition of fruit by-products. In the present study, it was found that neither Fibersol^®^, passion fruit, nor the interactions of both ingredients have a significant influence on the content of trypsin inhibitors, as it is explained in Section 2.4 of the present work.

It has been reported that the content of trypsin inhibitors significantly decreases after extrusion due to heat and strong mechanical stress [36]. In extruded formulations, the content of trypsin inhibitors varied between 0.22 and 0.33 TIU/mg in samples EF#6 and EF#7, respectively. These amounts of TIs are below the cited threshold limits (1–1.5 TIU/mg) [28], indicating that the extruded formulations are safe for human consumption. A significant reduction (*p* < 0.05) of TIA was observed, comparing extruded samples to their raw counterparts. This reduction varied between 86.7% (samples codified as #7) and 95.8% (samples codified as #2) (Table 2). This tendency was in accordance with the findings previously observed by other authors in different products based on pulses, such as lentils [4,37,38] or beans [39]. These facts show that extrusion allows the deactivation of trypsin inhibitors, which are thermally labile compounds whose structure is modified as a consequence of the application of high temperature, high pressure, and mechanical shear [40].

### 2.3. Antioxidant Activity

The antioxidant activity of formulations based on rice: chickpea was evaluated using three different in vitro direct assays (QUENCHER procedure). Specifically, the Folin–Ciocalteu, FRAP, and DPPH methods were carried out (Table 3).

In general, the highest values were obtained by applying the DPPH method. A consistent tendency that explains the extrusion effect on the antioxidant properties of formulations under study was not found. On one hand, the results obtained by the Folin–Ciocalteu method indicated that extrusion caused a significant decrease (*p* < 0.05) in the antioxidant activity of samples CE, EF#3, EF#5, EF#8, and EF#9, while in the others (EF#1, EF#2, EF#4, EF#6, and EF#7), no significant (*p* > 0.05) changes were observed after extrusion treatment. On the other hand, DPPH and FRAP assays tended to demonstrate an increase in the antioxidant activity, but this effect was not observed in the totality of the samples, with EF#2 presenting the highest activity through both methods (3.27 and 1.88 mg TE/g for DPPH and FRAP, respectively). Different results have been previously observed by other authors. For instance, Dilrukshi et al. (2022) reported that extrusion caused a decrease in the antioxidant activity of gluten-free snacks based on rice flour and fortified with cowpea and whey protein concentrate [6], while, by contrast, an increase in the antioxidant activity of wheat flour was found by Bangar et al. (2022) [41]. Other authors have primarily focused on the effect of incorporating additional ingredients, such as sour cherry pomace, into extruded gluten-free breads, which led to a fivefold increase in antioxidant activity through the ABTS method [42].

It is important to note that direct comparisons between this study’s findings and prior published data are challenging due to the unique characteristics of the sample analyzed, as well as the diversity of gluten-free products. These differences include variations in flour base (cereals, pseudocereals, and legumes), the enrichment products used (e.g., other legumes, fruits, fruit byproducts, vegetables, and algae), and the processing treatments applied (extrusion, germination, fermentation, or baking).

Regarding the extrusion effect on antioxidant properties, it is highly influenced by the food matrix composition. Various studies have demonstrated that the impact of extrusion on bioactive compounds varies significantly based on ingredient composition, processing conditions, and interactions among food components [43,44].

**Figure 1:**
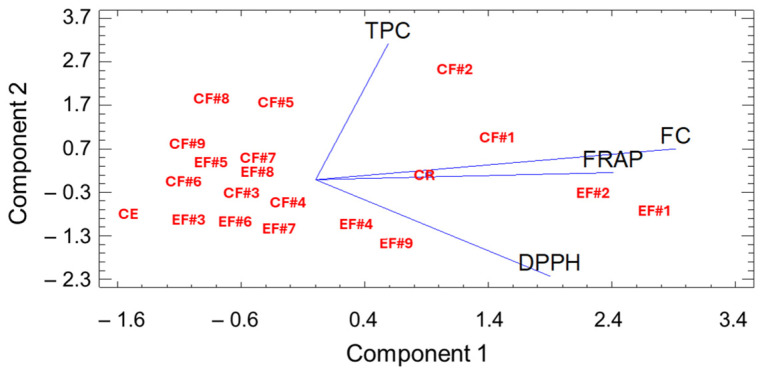
Principal Component Analysis (PCA) of Total Phenolic Content (TPC) of raw and extruded chickpea-rice-based flours and its in vitro antioxidant activity evaluated by Folin–Ciocalteau (FC), DPPH, and FRAP assays.

In order to enhance the study of the antioxidant activity of the analyzed samples, a principal component analysis (PCA) was performed. It was shown that the total phenolic content of the formulations is also intimately related to the antioxidant activity of the analyzed samples (Figure 1). PCA explained 63.09% of the total variation of the data, and it was performed considering the total phenolic content of raw and extruded chickpea-rice-based flours and its in vitro antioxidant activity evaluated by Folin–Ciocalteau, DPPH, and FRAP assays. Component 1 (characterized by antioxidant activity) showed 34.35% of the total variation of the data. On the other hand, component 2 (characterized by total phenolic content) explained 28.74% of the total variation of the data. These findings align with previous research indicating that extrusion may either enhance or degrade antioxidant activity depending on the extent of thermal processing, potential polymerization of phenolic compounds, and the release of bound phenolics [44]. Although some phenolic compounds are susceptible to heat degradation, extrusion can also enhance antioxidant potential by breaking down cell walls and increasing the bioavailability of phenolics, as observed in studies on extruded legumes [41]. The balance between these opposing effects is highly matrix-dependent, emphasizing the need for an optimized processing approach that maximizes the retention of bioactive compounds.

For a better understanding of the results obtained in the present study, it is important to highlight that the methods employed were in vitro assays, which may not directly translate to in vivo results; however, these results provide an approximation of the potential in vivo activity. Nevertheless, it would be interesting to carry out in vivo assays in the future to increase the knowledge about the level of antioxidant capacity in these types of formulations.

### 2.4. Effect of Ingredients on Chemical Composition of Analyzed Formulations

The results included in Table 4 showed that the incorporation of passion fruit and Fibersol^®^ had a significant effect on the chemical composition of gluten-free formulations analyzed in the present study (*p* ≤ 0.05). Although no significant effect attributable to the addition of passion fruit and Fibersol^®^ was observed on total phenolic content and trypsin inhibitor concentration, the presence of these individual ingredients or the combination of both significantly impacted the concentration of several bioactive compounds. The enrichment by incorporating Fibersol^®^ significantly affected the concentration of hydroxycinnamic acids (*p* = 0.0000), IP5 (*p* = 0.0001), IP6 (*p* = 0.0000), and total content of inositol phosphates (*p* = 0.0000). In addition, the incorporation of this ingredient (Fibersol^®^) also impacted the antioxidant properties determined by the DPPH (*p* = 0.0005) and FRAP (*p* = 0.0435) methods. Regarding the importance of adding passion fruit as a functional ingredient in gluten-free formulations based on rice and chickpea, it was observed that this component exhibited influence on the content of phenolic families (hydroxybenzoic acids: *p* = 0.0396; hydroxycinnamic acids: *p* = 0.0002; and flavonols: *p* = 0.0002), IP6 (*p* = 0.0321), total inositol phosphates (*p* = 0.0076), and antioxidant activity evaluated by Folin–Ciocalteu (*p* = 0.0000) and FRAP (*p* = 0.001) methods. Finally, significant interactions were found for passion fruit addition with Fibersol^®^ incorporation in the case of hydroxybenzoic acids, hydroxycinnamic acids, flavonols, IP5, IP6, total inositol phosphates content, and antioxidant activity measured by Folin and FRAP methods. These results bring to light the complexity of the analyzed formulations, whose composition is influenced not only by the presence of Fibersol^®^ and passion fruit but also by the interaction of both ingredients.

## 3. Materials and Methods

### 3.1. Formulated Flours Composition

Different gluten-free formulations (raw and extruded) were developed by mixing chickpea-rice (30:70) flours with different concentrations of Fibersol^®^ (Fibersol, Chicago, IL, USA) and passion-fruit-skin flour, referred to as passion fruit in this study (Table 5).

A Cyclone Mill (Udy Corp., Fort Collins, CO, USA) fitted with a 0.5-mm screen was used to reduce all formulated flours (from raw and extruded material) to uniform powders. After that, all formulated samples were stored in air-tight glass jars at room temperature until analyzed.

### 3.2. Extrusion Process Conditions

A twin-screw extruder Clextral EVOL HT32-H (Clextral, Inc., Tampa, FL, USA) was used for the extrusion processing of the samples. This equipment had six-barrel sections (each 128 mm in length) with co-rotating and intimately interlacing screws. The control formulation was a preliminary run under different extrusion processing conditions. These preliminary assays allowed us to determine the variables involved in the moisture content of the samples. The best conditions were applied to the formulations under study. These conditions consisted of a constant temperature of 140 ± 1 °C, a screw diameter (D) of 32 mm, a total screw length (L) of 768 mm, and an L/D ratio of 24. The variable-speed drive was 74.8 kW, Model ACS600 (ABB Automation, Inc., New Berlin, WI, USA). The screw speed was 500 rpm, and it was maintained constant. A combination of feeding, transporting, compression, and kneading elements was used to obtain a moderate-shear screw configuration (patent pending) [45]. The extrusion variables of screw speed, heating, and moisture addition were maintained constant for the processing of all formulations under study in order to allow to have as the main response variable, the different additions of the Fibersol^®^ and passion fruit skin.

The mixture was metered into the feed port at a rate of 20 kg/h (wwb) by using a twin-screw, loss-in-weight gravimetric feeder, Model LWFD5-20 (K-Tron Corp., Pitman, NJ, USA). A triplex variable stroke piston pump with 12mm plungers, Type VE-P33 (Bran and Luebbe, Wheeling, IL, USA), was used to deliver water to the extruder, with the aim of getting a final feed moisture content of 17%. The analyzed gluten-free formulations were subjected to extrusion through two circular dies (3.5 mm-diameter openings). After approximately 10 min, the formulations were recovered, the operation conditions of torque and pressure being constant.

### 3.3. Chemical Analysis

#### 3.3.1. Analysis of Total Phenolic Compounds and Phenolic Families

Total phenolic compounds as well as the phenolic families (hydroxybenzoic acids, hydroxycinnamic acids, and flavonols) were determined by QUENCHER (QUick, Easy, New, CHEap, and Reproducible) methodology [46,47]. This technique consists of submitting a small amount of the sample, previously homogenized until a particle size of 0.037 mm, to direct contact with the reagents of each determination. This methodology allows the quantification of both soluble and insoluble compounds; thus, it is possible to obtain a more accurate and reliable result.

##### Analysis of Total Phenolic Compounds by QUENCHER Methodology

The determination of total phenolic compounds was carried out by the Fast Blue BB method [48]. Briefly, 20 mg of each sample was weighed in triplicate and homogenized by vortex with 0.4 mL of 0.1% fast blue BB. Once samples were treated with the corresponding reagents, incubated (45 min in an orbital shaker), centrifuged (10 min at 6500 rpm), and filtered; the absorbance was determined at 420 nm (UV–Vis spectrometer, synergy HTX, Biotek), (Agilent Technologies, Sant Clara, CA, USA) The results were expressed as milligrams of gallic acid equivalent per gram (mg GAE/g, fw).

##### Analysis of Total Hydroxybenzoic Acids by QUENCHER Methodology

The procedure described by Bonoli et al. (2004) [49] was used to determine the content of hydroxybenzoic acids (HBC). For that, 10 mg of each sample was weighed in triplicate and properly treated before measuring the absorbance at 280 nm (UV–Vis spectrometer synergy HTX, Biotek). The results were expressed as milligrams of gallic acid equivalent per gram (mg GAE/g, fw).

##### Analysis of Total Hydroxycinnamic Acids by QUENCHER Methodology

The determination of hydroxycinnamic acids (HCC) was performed according to the procedure described by Bonoli et al. (2004) [49]. A total of 20 mg of each sample was weighed in triplicate and mixed with the corresponding reagents. After incubation (15 min in an orbital shaker), centrifugation (5 min at 6500 rpm), and filtration, the absorbance was determined at 320 nm (UV–Vis spectrometer, synergy HTX, Biotek). The results were expressed as milligrams of ferulic acid equivalent per gram (mg FAE/g, fw).

##### Analysis of Total Flavonols by QUENCHER Methodology

The determination of flavonols (FC) was carried out following the procedure proposed by Bonoli et al. (2004) [49]. For that, 20 mg of each sample was weighed in triplicate and mixed with the appropriate reagents. Next, samples were incubated (15 min in an orbital shaker), centrifuged (15 min at 6500 rpm), and filtered. Finally, the absorbance was determined at 370 nm employing a UV–Vis spectrometer, synergy HTX, and Biotek. The results were expressed in milligrams of quercetin equivalent per gram (mg QE/g, fw).

#### 3.3.2. Analysis of Non-Nutritional Factors

##### Analysis of Inositol Phosphates

In the present study, the identification and quantification of individual inositol phosphates (inositol tetraphosphate: IP4; inositol pentaphosphate: IP5; and inositol hexaphosphate: IP6) was carried out by the procedure described by Burbano et al. (1995) [50]. The sum of the individual compounds resulted in the total content of inositol phosphates. The methanolic extract of each sample was prepared and injected into the HPLC (Beckman System Gold Instrument, Los Angeles, CA, USA). A column consisting of a macroporous polymer PRP-1 (150 × 4.1 mm i.d., 5 “m) (Hamilton, Reno, NV, USA) was used. The chromatographic conditions were as follows: 45 °C temperature; mobile phase consisted of a mixture of methanol/water (51.4/48.5; *v*/*v*) with 0.8% of tetrabutylammonium hydroxide (40% in water), (Sigma, St. Louis, MO, USA), 0.1% of 5 M H_2_SO_4_, 0.05% of 91% formic acid (Sigma, St. Louis, MO, USA), and 100 μL of a phytic acid (6 mg mL^−1^); pH at 4.3, flow rate of 1 mL/min. A calibration curve was prepared from sodium phytate (Sigma, St. Louis, MO, USA). Results were expressed as mg of inositol phosphates per gram (mg/g, fw).

##### Analysis of Trypsin Inhibitors Activity

The analysis of trypsin inhibitor activity (TIA) was performed on the sample extracts using a small-scale quantitative assay proposed by Welham and Domoney (2000) [51]. For the determination of TIs, N-benzoyl-DL-arginine-p-nitroanilide hydrochloride (BAPNA) (Sigma, St. Louis, MO, USA) was employed as the trypsin substrate, and the values of trypsin inhibitor units (TIU) values were calculated from the absorbance read at 410 nm and expressed as mg per gram (mg/g, fw).

#### 3.3.3. Antioxidant Activity

Three different in vitro direct methods (QUENCHER procedure), specifically Folin–Ciocalteu, FRAP, and DPPH, were applied to evaluate the antioxidant activity of gluten-free formulations under study.

##### Folin–Ciocalteu Assay

The Folin–Ciocalteu assay was carried out according to the procedure described by del Pino-García et al. (2015) [46]. For that, 15 mg of each sample was weighed in triplicate and properly treated before measuring the absorbance at 750 nm (UV–Vis spectrometer Synergy HTX, Biotek). The results were expressed as milligrams of gallic acid equivalent per gram of sample (mg GAE/g, fw).

##### FRAP Assay

The evaluation of the ferric-reducing power by the samples was performed following the procedure described by del Pino-García et al. (2015) [46]. In brief, 15 mg of each sample (weighed in triplicate) was mixed with 10 mL of FRAP reagent, homogenized by vortex, incubated (37 °C, 30 min, continuous agitation), centrifuged (5 min at 7000 rpm), and filtrated. The absorbance was recorded at 595 nm (UV–Vis spectrometer synergy HTX, Biotek). The results were expressed as milligrams of TROLOX equivalent per gram of sample (mg TE/g, fw).

##### DPPH Assay

The procedure described by del Pino-García et al. (2015) [46] was applied to determine the scavenging capacity of the 2,2-diphenyl-1-picriylhydrazyl (DPPH) radical by the sample. Then, 15 mg was weighed in triplicate and mixed with 10 mL of DPPH reagent. After homogenization by vortex, incubation (1 h, orbital shaking), centrifugation (5 min at 7000 rpm), and filtration, the absorbance was measured at 517 nm (UV–Vis spectrometer, synergy HTX, Biotek). The results were expressed as milligrams of TROLOX equivalent per gram of sample (mg TE/g, fw).

### 3.4. Statistical Analysis

Mean ± standard deviations (SD) were determined, and the data were statistically analyzed by Analysis of Variance (ANOVA) in order to find differences between samples subjected to the same treatment (untreated or extruded). To compare the same formulations between and after extrusion, a Student’s *t*-test was carried out. To explore the impact of the ingredients incorporated into the formulations for their enrichment (passion fruit and Fibersol^®^), two-way interactions were included in the ANOVA. A Duncan test was performed to find the differences. All statistical analyses were carried out using Statgraphics Plus 5.1 software. The statistical significance level was set at *p* < 0.05. Principal Component Analysis was performed to establish relationships between total phenolic content and in vitro antioxidant activity between samples under study.

## 4. Conclusions

Attending to the current tendency found in society consisting of the search for new food products, nutritious, sustainable, and with functional properties, novel extruded formulations based on rice-chickpea flours and fortified with different percentages of Fibersol^®^ and passion-fruit-skin flour were developed and characterized in terms of their phytochemical composition. It was demonstrated that extrusion cooking is an appropriate technology to maintain the phenolic compounds present in this type of food product while effectively reducing the content of non-nutritional factors such as trypsin inhibitors. The antioxidant activity of formulations was evaluated in vitro, and it was observed that after extrusion treatment, the antioxidant properties were preserved. Moreover, the impact of the ingredients added to the formulation’s enrichment (passion fruit and Fibersol^®^) was studied, and the obtained results brought to light the complexity of the analyzed formulations, whose composition is influenced not only by the presence of Fibersol^®^ and passion fruit but also by the interaction of both ingredients. Moreover, it was evidenced that the addition of byproducts, such as passion fruit, enhanced the composition of different formulations, and it could be interesting to further explore the incorporation of similar byproducts to assess their potential benefits. In future studies, it would be valuable to evaluate the biological activities of the formulations, particularly their antioxidant capacity, through in vivo methodologies to obtain results that more accurately reflect real conditions. Additionally, once these formulations are fully characterized in terms of their nutritional and functional aspects, the study of their sensorial characteristics would be an interesting aspect to explore consumer acceptance of these products.

## Figures and Tables

**Table 1:** Profile of phenolic compounds of raw and extruded chickpea-rice-based flours, determined by QUENCHER methodology (mean ± SD).

Sample	HBC (mg GAE/g)	HCC (mg FAE/g)	FC (mg QE/g)	TPC (mg GAE/g)
CR	4.08 ± 0.40^d,B^	0.59 ± 0.06^f,B^	0.140 ± 0.013^g,B^	12.34 ± 0.26^e,B^
CE	1.62 ± 0.15^a,A^	0.34 ± 0.02^c,A^	0.084 ± 0.008^a,A^	10.26 ± 0.31^d,A^
CF#1	4.88 ± 0.29^e,B^	0.44 ± 0.04^e,B^	0.091 ± 0.006^cd,A^	11.40 ± 0.08^d,B^
EF#1	2.17 ± 0.14^b,A^	0.29 ± 0.03^b,A^	0.125 ± 0.011^d,B^	8.29 ± 0.78^c,A^
CF#2	5.35 ± 0.11^f,B^	0.34 ± 0.03^bc,B^	0.067 ± 0.007^a,A^	14.28 ± 0.07^f,B^
EF#2	2.32 ± 0.13^b,A^	0.22 ± 0.02^a,A^	0.102 ± 0.010^bc,B^	11.30 ± 0.90^e,A^
CF#3	5.31 ± 0.25^f,B^	0.40 ± 0.04^de,B^	0.078 ± 0.007^ab,A^	8.53 ± 0.40^a,B^
EF#3	1.81 ± 0.09^a,A^	0.23 ± 0.02^a,A^	0.098 ± 0.009^bc,B^	7.44 ± 0.31^b,A^
CF#4	2.66 ± 0.15^c,B^	0.38 ± 0.04^cde,B^	0.090 ± 0.009^cd,A^	9.51 ± 0.50^bc,B^
EF#4	2.19 ± 0.07^b,A^	0.24 ± 0.02^a,A^	0.092 ± 0.008^ab,A^	6.32 ± 0.33^a,A^
CF#5	1.91 ± 0.10^a,A^	0.31 ± 0.03^ab,B^	0.092 ± 0.005^bc,A^	13.83 ± 0.49^f,B^
EF#5	2.35 ± 0.05^b,B^	0.21 ± 0.02^a,A^	0.094 ± 0.007^ab,A^	9.80 ± 0.50^d,A^
CF#6	4.12 ± 0.32^d,B^	0.28 ± 0.03^a,B^	0.070 ± 0.006^a,A^	8.97 ± 0.05^ab,B^
EF#6	1.70 ± 0.15^a,A^	0.22 ± 0.02^a,A^	0.110 ± 0.009^c,B^	6.59 ± 0.44^a,A^
CF#7	2.53 ± 0.12^bc,B^	0.44 ± 0.04^e,B^	0.120 ± 0.011^f,B^	9.70 ± 0.22^c,B^
EF#7	2.31 ± 0.08^b,A^	0.27 ± 0.02^b,A^	0.101 ± 0.009^bc,A^	6.53 ± 0.38^a,A^
CF#8	2.22 ± 0.05^ab,B^	0.36 ± 0.03^bcd,A^	0.100 ± 0.009^de,A^	13.84 ± 0.68^f,B^
EF#8	1.80 ± 0.22^a,A^	0.35 ± 0.02^c,A^	0.138 ± 0.011^e,B^	10.18 ± 0.27^d,A^
CF#9	2.15 ± 0.03^a,A^	0.32 ± 0.03^ab,B^	0.106 ± 0.010^e,A^	9.21 ± 0.53^bc,B^
EF#9	3.94 ± 0.17^c,B^	0.28 ± 0.03^b,A^	0.110 ± 0.010^c,A^	7.99 ± 0.04^bc,A^

In each column, statistically significant differences (*p* < 0.05) between all identically processed samples (untreated or subjected to extrusion), compared by the Duncan test, are shown by using a small superscript letter, whereas statistically significant differences (*p* < 0.05) due to extrusion cooking for the same formulation, compared by Student’s *t*-test, are shown by using a capital superscript letter. HBC: hydroxybenzoic acids content; HCC: hydroxycinnamic acids content; FC: flavonol content; TPC: total phenolic content; GAE: gallic acid equivalent; FAE: ferulic acid equivalent; QE: quercetin equivalent. CR: control raw formulation; CE: control extruded formulation; CF: control formulation (raw); EF: extruded formulation.

**Table 2:** The inositol phosphate content (mg/g) and trypsin inhibitory activity (TIU/mg) of raw and extruded chickpea-rice-based flours (mean ± SD).

Sample	IP4	IP5	IP6	Total IP	Trypsin Inhibition
CR	0.26 ± 0.01^a,A^	0.49 ± 0.01^de,A^	2.19 ± 0.27^c,A^	2.95 ± 0.27^c,A^	5.86 ± 0.16^e,B^
CE	0.27 ± 0.01^bc,A^	0.54 ± 0.09^d,A^	2.82 ± 0.11^f,B^	3.52 ± 0.22^e,B^	0.31 ± 0.02^de,A^
CF#1	0.26 ± 0.01^a,A^	0.48 ± 0.03^cde,A^	2.24 ± 0.34^c,A^	2.97 ± 0.38^c,A^	5.89 ± 0.19^e,B^
EF#1	0.26 ± 0.01^ab,A^	0.51 ± 0.04^bcd,A^	1.94 ± 0.11^d,A^	2.71 ± 0.07^c,A^	0.29 ± 0.01^cd,A^
CF#2	0.26 ± 0.02^a,A^	0.46 ± 0.03^bcd,A^	2.23 ± 0.13^c,B^	2.94 ± 0.12^c,B^	6.21 ± 0.27^f,B^
EF#2	0.28 ± 0.01^c,A^	0.48 ± 0.02^abcd,A^	1.90 ± 0.04^d,A^	2.66 ± 0.07^c,A^	0.26 ± 0.02^b,A^
CF#3	0.25 ± 0.01^a,A^	0.51 ± 0.03^e,A^	2.15 ± 0.21^c,A^	2.91 ± 0.20^c,A^	4.40 ± 0.08^d,B^
EF#3	0.26 ± 0.01^ab,A^	0.40 ± 0.27^cd,A^	1.66 ± 0.57^e,A^	2.32 ± 0.72^d,A^	0.30 ± 0.02^d,A^
CF#4	0.26 ± 0.00^a,A^	0.43 ± 0.02^ab,A^	1.65 ± 0.06^b,A^	2.33 ± 0.06^b,A^	2.50 ± 0.11^a,B^
EF#4	0.25 ± 0.00^a,A^	0.50 ± 0.08^abcd,A^	1.55 ± 0.13^c,A^	2.30 ± 0.19^b,A^	0.30 ± 0.02^d,A^
CF#5	0.25 ± 0.00^a,A^	0.41 ± 0.02^a,A^	1.75 ± 0.09^b,B^	2.41 ± 0.07^b,A^	2.47 ± 0.23^a,B^
EF#5	0.25 ± 0.00^a,A^	0.47 ± 0.07^abcd,A^	1.56 ± 0.03^c,A^	2.28 ± 0.09^b,A^	0.28 ± 0.00^c,A^
CF#6	0.26 ± 0.00^a,A^	0.42 ± 0.03^ab,A^	1.84 ± 0.17^b,A^	2.52 ± 0.20^b,A^	2.77 ± 0.09^b,B^
EF#6	0.25 ± 0.00^ab,A^	0.45 ± 0.03^abc,A^	1.85 ± 0.18^d,A^	2.55 ± 0.18^c,A^	0.22 ± 0.01^aA^
CF#7	0.26 ± 0.01^a,A^	0.41 ± 0.05^a,A^	1.37 ± 0.07^a,B^	2.03 ± 0.08^a,B^	2.48 ± 0.09^a,B^
EF#7	0.26 ± 0.00^ab,A^	0.45 ± 0.04^abc,A^	0.98 ± 0.08^a,A^	1.68 ± 0.08^a,A^	0.33 ± 0.01^e,A^
CF#8	0.26 ± 0.00^a,A^	0.41 ± 0.02^a,A^	1.83 ± 0.14^b,B^	2.50 ± 0.12^b,B^	3.58 ± 0.30^c,B^
EF#8	0.26 ± 0.02^ab,A^	0.43 ± 0.05^ab,A^	1.25 ± 0.04^b,A^	1.87 ± 0.18^a,A^	0.31 ± 0.02^de,A^
CF#9	0.27 ± 0.01^a,A^	0.44 ± 0.02^abc,A^	1.83 ± 0.18^b,B^	2.54 ± 0.20^b,B^	3.69 ± 0.33^c,B^
EF#9	0.26 ± 0.02^ab,A^	0.41 ± 0.02^a,A^	1.54 ± 0.05^c,A^	2.21 ± 0.07^b,A^	0.26 ± 0.01^b,A^

In each column, statistically significant differences (*p* < 0.05) between all identically processed samples (untreated or subjected to extrusion), compared by the Duncan test, are shown by using a small superscript letter, whereas statistically significant differences (*p* < 0.05) due to extrusion cooking for the same formulation, compared by Student’s *t*-test, are shown by using a capital superscript letter. IP4: inositol tetraphosphate; IP5: inositol pentaphosphate; IP6: inositol hexaphosphate; Total IP: total content of inositol phosphates. CR: control raw formulation; CE: control extruded formulation; CF: control formulation (raw); EF: extruded formulation.

**Table 3:** In vitro antioxidant activity of raw and extruded chickpea-rice-based flours (mean ± SD) determined by QUENCHER procedure.

Sample	Folin–Ciocalteu (mg GAE/g)	DPPH Radical Scavenging Capacity (mg TE/g)	FRAP (mg TE/g)
CR	1.104 ± 0.040^d,B^	3.199 ± 0.162^f,A^	0.879 ± 0.059^a,B^
CE	0.554 ± 0.039^a,A^	3.014 ± 0.123^d,A^	0.618 ± 0.010^a,A^
CF#1	1.370 ± 0.074^e,A^	2.648 ± 0.122^d,A^	1.115 ± 0.108^c,A^
EF#1	1.464 ± 0.068^d,A^	3.133 ± 0.068^de,B^	1.464 ± 0.074^e,B^
CF#2	1.078 ± 0.091^d,A^	1.848 ± 0.101^a,A^	1.733 ± 0.023^e,A^
EF#2	0.974 ± 0.043^c,A^	3.270 ± 0.072^e,B^	1.879 ± 0.057^g,B^
CF#3	0.937 ± 0.093^c,B^	2.361 ± 0.120^c,A^	0.921 ± 0.034^ab,A^
EF#3	0.728 ± 0.067^b,A^	2.588 ± 0.026^c,B^	0.974 ± 0.094^b,A^
CF#4	0.825 ± 0.073^abc,A^	2.969 ± 0.110^e,B^	0.923 ± 0.060^ab,A^
EF#4	0.908 ± 0.072^c,A^	2.564 ± 0.036^c,A^	1.471 ± 0.098^e,B^
CF#5	0.867 ± 0.013^abc,B^	2.208 ± 0.084^bc,A^	1.067 ± 0.094^bc,A^
EF#5	0.697 ± 0.056^b,A^	2.154 ± 0.052^a,A^	1.344 ± 0.011^de,B^
CF#6	0.808 ± 0.052^ab,A^	2.295 ± 0.075^c,A^	0.888 ± 0.057^a,A^
EF#6	0.866 ± 0.071^c,A^	2.282 ± 0.148^ab,A^	1.068 ± 0.088^b,B^
CF#7	0.761 ± 0.066^a,A^	2.202 ± 0.238^bc,A^	1.287 ± 0.113^d,A^
EF#7	0.871 ± 0.061^c,A^	2.341 ± 0.080^b,A^	1.246 ± 0.060^cd,A^
CF#8	0.816 ± 0.024^ab,B^	1.995 ± 0.049^ab,B^	1.009 ± 0.023^abc,A^
EF#8	0.743 ± 0.036^b,A^	2.366 ± 0.150^b,A^	1.203 ± 0.016^c,B^
CF#9	0.882 ± 0.009^bc,B^	1.787 ± 0.028^a,A^	1.048 ± 0.078^bc,A^
EF#9	0.682 ± 0.025^b,A^	3.039 ± 0.116^d,B^	1.710 ± 0.135^f,B^

In each column, statistically significant differences (*p* < 0.05) between all samples identically processed (untreated or subjected to extrusion), compared by the Duncan test, are shown by using a small superscript letter, whereas statistically significant differences (*p* < 0.05) due to extrusion cooking for the same formulation, compared by Student’s *t*-test, are shown by using a capital superscript letter. GAE: gallic acid equivalent; TE: TROLOX equivalent. CR: control raw formulation; CE: control extruded formulation; CF: control formulation (raw); EF: extruded formulation.

**Table 4:** Report of *p*-values table of ANOVA regarding the main outcomes and double interactions due to the content of Fibersol^®^ and Passion fruit on bioactive compounds, non-nutritional factors, and antioxidant activity of fortified gluten-free formulations.

	Fibersol^®^ Content	Passion Fruit Content	Fibersol^®^ Content x Passion Fruit Content
**Total phenolic content**	0.2224	0.5953	0.7507
**Hydroxybenzoic acids**	0.8337	**0.0396**	**0.0209**
**Hydroxycinnamic acids**	**0.0000**	**0.0002**	**0.0000**
**Flavonols**	0.3467	**0.0002**	**0.0001**
**IP4**	0.3572	0.6945	0.8870
**IP5**	**0.0001**	0.2849	0.4188
**IP6**	**0.0000**	**0.0321**	**0.0001**
**Total IP**	**0.0000**	**0.0076**	**0.0000**
**Trypsin inhibition**	0.1918	0.2678	0.2741
**Folin–Ciocalteu**	0.2319	**0.0000**	**0.0000**
**DPPH**	**0.0005**	0.7130	0.7738
**FRAP**	**0.0435**	**0.0001**	**0.0027**

Emboldened numbers indicate significant effects at *p* ≤ 0.05. IP4: inositol tetraphosphate; IP5: inositol pentaphosphate; IP6: inositol hexaphosphate; Total IP: total content of inositol phosphates; DPPH: 2,2-diphenyl-1-picriylhydrazyl; FRAP: ferric reducing antioxidant power.

**Table 5:** The composition of chickpea-rice flour formulations was analyzed.

	Sample	Characteristics		
		% Mixture CP:R	% Passion Fruit	% Fibersol^®^
**CR**	Control raw flour	93.75	0	0
**CE**	Control extruded flour			
**CF#1**	Control formulation 1	68.75	20	5
**EF#1**	Extruded formulation 1			
**CF#2**	Control formulation 2	76.25	12.5	5
**EF#2**	Extruded formulation 2			
**CF#3**	Control formulation 3	83.75	5	5
**EF#3**	Extruded formulation 3			
**CF#4**	Control formulation 4	66.25	20	7.5
**EF#4**	Extruded formulation 4			
**CF#5**	Control formulation 5	73.75	12.5	7.5
**EF#5**	Extruded formulation 5			
**CF#6**	Control formulation 6	81.25	5	7.5
**EF#6**	Extruded formulation 6			
**CF#7**	Control formulation 7	63.75	20	10
**EF#7**	Extruded formulation 7			
**CF#8**	Control formulation 8	71.25	12.5	10
**EF#8**	Extruded formulation 8			
**CF#9**	Control formulation 9	78.75	5	10
**EF#9**	Extruded formulation 9			

CP:R = mixture of 30% chickpea with 70% rice flour.

## Data Availability

The original contributions presented in this study are included in the article. Further inquiries can be directed to the corresponding author.
